# Case Report: *Clostridium neonatale* Bacteremia in a Preterm Neonate With Necrotizing Enterocolitis

**DOI:** 10.3389/fped.2021.771467

**Published:** 2021-12-02

**Authors:** Nadim Cassir, Isabelle Grandvuillemin, Manon Boxberger, Priscilla Jardot, Farid Boubred, Bernard La Scola

**Affiliations:** ^1^Department of Clinical Microbiology, Aix Marseille Université, Institut de Recherche pour le Développement, Assistance Publique des Hôpitaux de Marseille, Microbes, Evolution, Phylogénie et Infection, Marseille, France; ^2^Institut Hospitalo-Universitaire Méditerranée Infection, Marseillle, France; ^3^Department of Neonatology, Hôpital La Conception, Assistance Publique des Hôpitaux de Marseille, AMU, Marseille, France; ^4^Neonatal Unit, C2 VN, Hospital University La Conception, Assistance Publique des Hôpitaux de Marseille, AMU, Marseille, France

**Keywords:** case report, necrotizing enterocolitis, preterm neonate, *Clostridium neonatale*, gut microbiota

## Abstract

Necrotizing enterocolitis is a life-threatening acquired gastrointestinal disorder among preterm neonates and is associated with a high mortality rate and long-term neurodevelopmental morbidity. No etiologic agent has been definitively established; nonetheless, the most implicated bacteria include members of the *Clostridium* genus. We reported here on a case of *Clostridium neonatale* bacteremia in a preterm neonate with necrotizing enterocolitis, providing more information regarding the potential role of this bacterium in pathogenesis of necrotizing enterocolitis. We emphasized the sporulating form of *C. neonatale* that confers resistance to disinfectants usually applied for the hospital environmental cleaning. Further works are needed to establish the causal relationship between the occurrence of NEC and the isolation of *C. neonatale*, with promising perspectives in terms of diagnostic and therapeutic management.

## Introduction

Necrotizing enterocolitis is a life-threatening acquired gastrointestinal disorder among preterm neonates, especially those of very low birth weight (<1,500 g) for which the incidence reaches 7% (1). Necrotizing enterocolitis (NEC) is associated with a high mortality rate (15–30%) and long-term neurodevelopmental morbidity (1). Although NEC pathophysiology remains unclear, prematurity, enteral feeding strategies, gut bacterial colonization, and inappropriate proinflammatory response are major factors involved in NEC development (1). Gut dysbiosis is considered the cornerstone of NEC pathogenesis. One of the main factors leading to gut dysbiosis is pre- and post-natal antibiotic exposure (1).

There is evidence of the role of specific bacteria in the pathogenesis of NEC. Indeed, outbreaks of NEC have long been described, although the reported causal agents greatly differed (1). *Pneumatosis intestinalis* is one of the specific radiological signs of NEC that likely represents submucosal gas produced by bacterial fermentation (2). In addition, it has been shown that NEC could not be reproduced in germ-free animals and rarely occurs until at least 8–10 days postpartum in preterm neonates, after strict anaerobic bacterial populations establishment (2).

We report here on a case of *Clostridium neonatale* bacteremia in a preterm neonate with necrotizing enterocolitis providing more information regarding the potential role of this bacterium in the pathogenesis of NEC.

## Case

**Figure 1:**
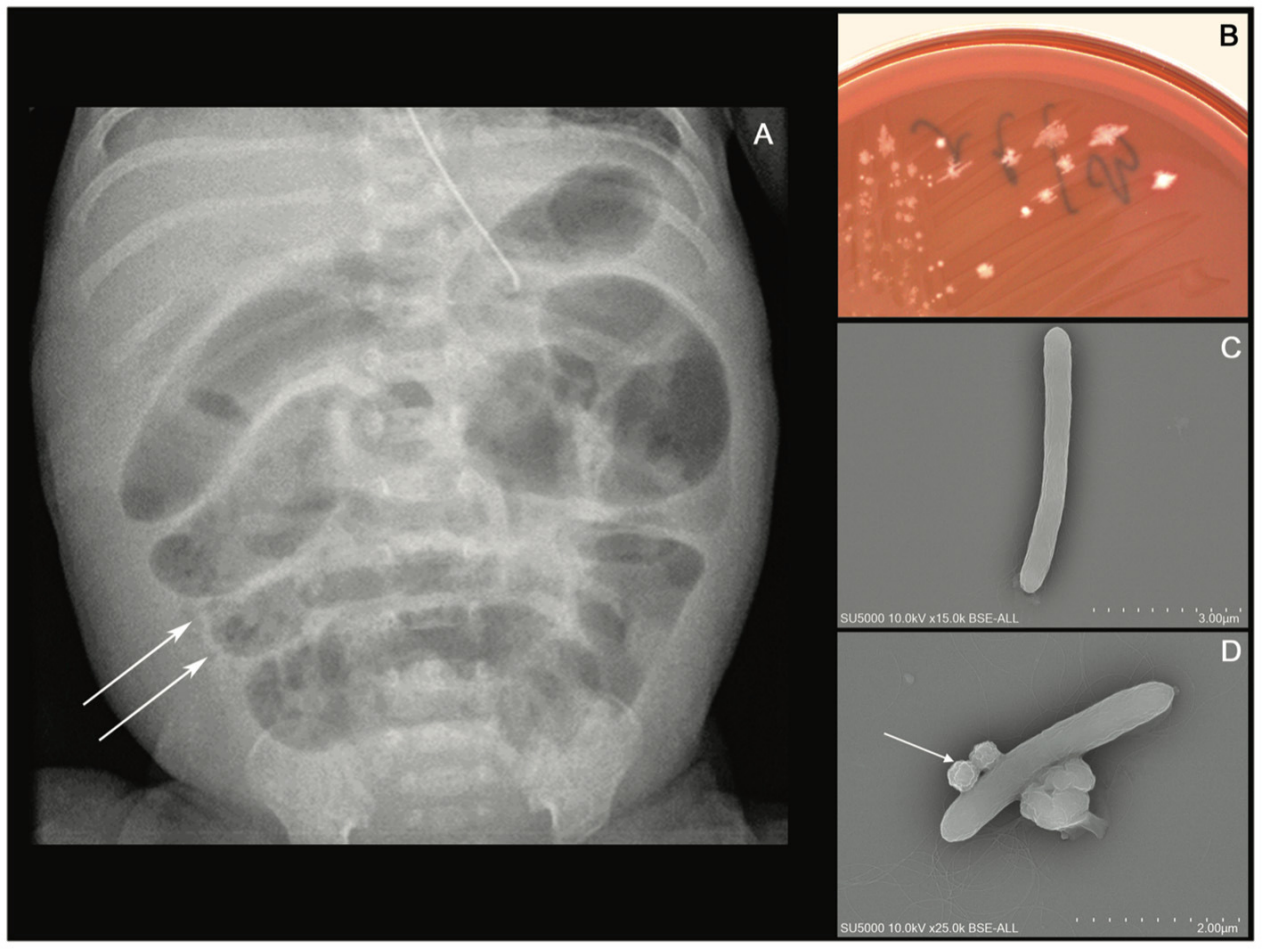
**(A)** Abdominal X-ray showing intramural bowel gas also known as *pneumatosis intestinalis*, **(B)** culture growth of cream-colored colonies on COS after 24 h of incubation at 37°C in the anaerobic chamber, **(C)** *Clostridium neonatale* rod-shaped cells using Hitachi SU5000 scanning electron microscope. Scale bar and acquisition settings are shown on the original micrograph, and **(D)** sporulating *C. neonatale* cell.

**Figure 2:**
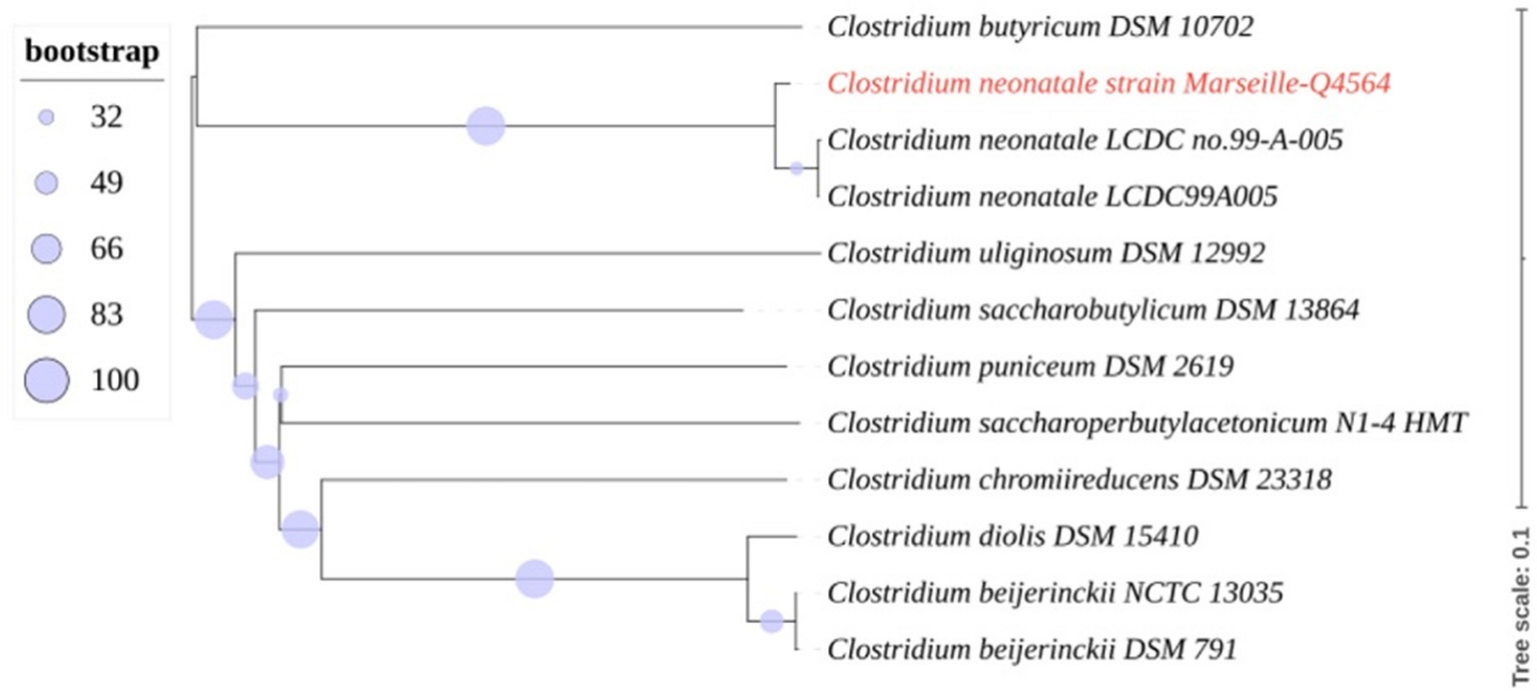
Whole-genome (nucleotides) based phylogenetic tree highlighting the position of *C. neonatale* strain Marseille-Q4564 relative to other closely related bacterial taxa.

A 24^+4^-week-old gestational age female infant, with a birth weight of 800 g (95th percentile), was diagnosed with severe necrotizing enterocolitis (NEC) at the day of life (DoL) 21. She was born after cesarean delivery for preterm premature rupture of membranes and after antenatally completing a course of betamethasone. She was immediately admitted to our neonatal intensive care unit (NICU) and required exogenous surfactant combined with nasal continuous positive airway pressure for respiratory distress syndrome. The initial intravenous antibiotics (ampicillin and gentamycin) administered for suspected neonatal infection were stopped on DoL 2. Own breastfeeding was started from DoL1 (colostrum) and was well tolerated. On DoL 21, clinical signs of feeding intolerance suddenly occurred with bilious gastric residuals, abdominal distention, and rectal bleeding. Abdominal X-ray showed *pneumatosis intestinalis* (Figure 1A). White blood cells count, and C-reactive protein (CRP) respectively peaked at 40 × 10^9^ /L (normal value range: 4.5 to 11 × 10^9^ /L) and 240 mg/L (normal value <5 mg/L). Enteral feeding was stopped and broad-spectrum antibiotics with vancomycin, cefotaxime, amikacin, and metronidazole were initiated. The yield of blood cultures allowed for the isolation of *Clostridium neonatale*. More specifically, 24 h after blood sampling, one anaerobic bottle out of two blood culture sets tested positive. Colony growth was observed on Columbia agar medium (Oxoid, Dardilly, France) supplemented with 5% (vol/vol) sheep blood and incubated for 24 h at 37°C in an anaerobic chamber (80%N2, 10% CO2, and 10% H2) (AES Chemunex, Bruz, France). Colonies were cream-colored and opaque after 24 h of growth (Figure 1B). By electronic microscopy using Hitachi SU5000 scanning electron microscope (Hitachi High-Tech Corporation, Tokyo, Japan), colonies were bacilli (Figure 1C). Bacterial cells were Gram-positive (not shown). Sporulation was observed after 24 h under aerobic conditions (Figure 1D). Using matrix-assisted laser desorption ionization-time of flight (MALDI-TOF) mass spectrometry Vitek MS (bioMérieux, France), the organism was identified as *Clostridium* spp., and this was confirmed as *C. neonatale* through 16S rRNA PCR and whole-genome sequencing. In brief, strain Marseille-Q4564 exhibited a 99.66% 16S rRNA sequence similarity with *C*.*neonatale*^T^ (Accession number AF275949.1), the phylogenetically closest bacterium standing in nomenclature. Furthermore, a digital DNA–DNA hybridization revealed a maximum identity similarity of only 91%, and an OrthoANI parameter provided values of 98.89 and 98.95%, respectively, between Marseille-Q4564 and *C*.*neonatale*^T^ (NZ_PDCJ01000001.1 and NZ_LN890312.1). Taken together, these results confirm the status of this strain belonging to the *C. neonatale* species (Figure 2).

Antimicrobial susceptibility testing was performed. Minimum Inhibitory Concentration (MIC) of tigecycline and vancomycin were determined using Etest (Biomérieux, France), while imipenem, metronidazole, and clindamycin MICs were obtained by the disk diffusion method using SIRscan (i2a diagnostics, France). Strain Marseille-Q4564 was susceptible to all the above-mentioned antibiotics tested.

While stool culture under anaerobic conditions after a heat shock was negative, specific RT-PCR of *C. neonatale* tested on stools was positive (3).

Because of a sub-occlusive digestive syndrome with systemic inflammation, surgery was performed 7 days after NEC (DoL28). A severe ischemic jejunum injury was found and was treated with a conservative ileostomy. Culture and RT-PCR specific for *C. neonatale* tested on the intestine fragment were negative. Cefotaxime and metronidazole were continued for 2 weeks post-surgery. Prolonged parenteral nutrition with minimal enteral feeding was required and preserved adequate growth and a good outcome at 6 months follow-up.

## Discussion

Regarding necrotizing enterocolitis (NEC), several causative microorganisms have been proposed. Nonetheless, the most implicated bacteria include members of the *Clostridium* genus, especially *Clostridium butyricum, C. perfringens, C. paraputrificum*, and *C. neonatale*. Indeed, *C. butyricum* has frequently been recovered from biological samples of premature neonates suffering from NEC (4). Additionally, in quail and chicken animal models of NEC, *C. butyricum, C. perfringens, and C. paraputrificum* were shown to be responsible for NEC-like lesions (5). *C. neonatale* and *C. butyricum* were reported to be significantly overrepresented among colonic mucosal samples from premature piglets with NEC (6). In 2002, an outbreak of NEC occurred in a Canadian neonatal intensive care unit (7). Blood cultures from three out of six premature neonates grew the same strain further described as *C. neonatale*, belonging to cluster I of the *Clostridium* genus sensu stricto (8). Another research team that performed microbiota analysis in 16 cases and 78 controls showed an association between the isolation of *Clostridium neonatale* from the stools of preterm neonates and the occurrence of NEC (9). A specific *rpoB*-based quantitative real-time PCR has been recently designed and developed to detect *C. neonatale* directly from the stool specimens of patients. In a case-control study, *C. neonatale* was significantly more prevalent in stools from preterm neonates with NEC than in controls (respectively 30/88 (34%) vs. 9/71 (13%); *p* =.003) (3). By whole-genome sequencing, genes encoding the secretion of bacterial toxins, especially hemolysins and *C. difficile* toxins A/B (TcdA/B) had been previously found (10). These latter are involved in the pathogenic mechanism of *C. difficile* colitis, which is the consequence of TcdA (enterotoxin) and TcdB (cytotoxin) production inducing colonic tissue damage (1).

The preterm neonate acquires its microbiota within the confines of the NICU where colonization is influenced by iatrogenic manipulations including the hospital environment. Recently, an outbreak of NEC was epidemiologically linked to sporulating *C. butyricum* contamination within the hospital (11). Knowing that spore-forming bacteria are highly resistant to usual hospital disinfectants, sporicidal agents should be used for environmental cleaning in case of sporulating *C. neonatale* infection. Therefore, we hypothesized that enteric isolation precautions like those applied for patients with *C. difficile* colitis may be effective in controlling NEC outbreaks.

Perinatal exposure to antibiotics has been identified as a risk factor for NEC, in particular when administered more than 5 days (12). The postnatal antibiotic exposure, in this case, may have increased the risk of NEC. Moreover, in this case, the use of large spectrum antibiotics during the episode of NEC may have participated in the secondary worsening 7 days after the initial diagnosis of NEC, by promoting gut dysbiosis. The length of treatment and choice of antimicrobial agents for presumed and proven episodes of NEC vary among centers due to a lack of supportive evidence and guidelines. The study of Cantey et al. showed recently that the implementation of antibiotic stewardship programs including appropriate dosages and narrow-spectrum regimens was effective in reducing unnecessary antibiotic use in NICUs (13). In addition, probiotics have been extensively studied to mitigate gut dysbiosis in preterm neonates and prevent the occurrence of NEC (14). A low to moderate level of certainty about the effects of probiotic supplementation on the risk of NEC has been reported. To date, product safety and quality remain of concern especially in a vulnerable population such as preterm neonates. Therefore, probiotics are not routinely used in our NICU. In this case, we cannot rule out the possibility that the administration of a combination of different strains of probiotics may have prevented the occurrence of NEC. Further works are needed to address this issue.

Cultivation of strictly anaerobic species is challenging. Only a few studies analyzing the gut bacterial profile associated with NEC have used strategies to optimize the growth of strictly anaerobic bacteria such as heat shock for sporulating anaerobes, direct inoculation in anaerobic culture bottles, selective culture media, and the use of an anaerobic chamber (14). However, it is too early to state that routine microbiological exams in preterm neonates with NEC should include bacterial culture under anaerobic conditions and RT-PCR testing specific for *C. neonatale, C. butyricum*, and *C. perfringens* on a stool, blood, and surgical samples. This case of *Clostridium neonatale* bacteremia in a preterm neonate with necrotizing enterocolitis provides more information regarding the potential role of this bacterium in the pathogenesis of necrotizing enterocolitis. We hypothesize that the pathophysiological mechanism of *C. neonatale* may be like that of toxigenic *C. difficile* in adults with pseudomembranous colitis. Further works are needed to establish the causal relationship between the occurrence of NEC and the isolation of *C. neonatale* (15), with promising perspectives in terms of diagnostic and therapeutic management.

## Data Availability Statement

The datasets presented in this study can be found in online repositories. The names of the repository/repositories and accession number(s) can be found below: https://www.ncbi.nlm.nih.gov/genbank/, NZ_PDCJ01000001.1.

## Ethics Statement

Ethical review and approval was not required for the study on human participants in accordance with the local legislation and institutional requirements. Written informed consent to participate in this study was provided by the participant's legal guardian/next of kin.

## Author Contributions

NC: conceptualized and designed the study, drafted the initial manuscript, and reviewed and revised the manuscript. IG, MB, and PJ: designed the data collection instruments, collected data, carried out the initial analyses, reviewed, and revised the manuscript. BL and FB: conceptualized and designed the study, supervised data collection, performed analysis and interpretation of data, and critically reviewed the manuscript for important intellectual content. All authors approved the final manuscript as submitted and agree to be accountable for all aspects of the work.

## Funding

This research was supported by the French Government under the Investissements d'avenir (investments for the future) program managed by the Agence Nationale de la Recherche grant number [10-IAHU-03].

## Conflict of Interest

The authors declare that the research was conducted in the absence of any commercial or financial relationships that could be construed as a potential conflict of interest.

## Publisher's Note

All claims expressed in this article are solely those of the authors and do not necessarily represent those of their affiliated organizations, or those of the publisher, the editors and the reviewers. Any product that may be evaluated in this article, or claim that may be made by its manufacturer, is not guaranteed or endorsed by the publisher.

## Abbreviations

NEC: necrotizing enterocolitis
DoL: days of life
CRP: C-reactive protein.
